# Feeding Models in Classical Phenylketonuria: Do They Make a Difference in Infant Sleep?

**DOI:** 10.3390/nu17183022

**Published:** 2025-09-22

**Authors:** Ezgi Burgaç, Ebru Çiçek Türköz, Adnan Barutçu, Fatma Derya Bulut, Deniz Kor, Tuğçe Kartal, Sema Uzunoğlu, Esra Kara, Burcu Köseci, İrem Kaplan, Nazlı Totik, Neslihan Onenli Mungan

**Affiliations:** 1 Department of Pediatric Metabolism, Cukurova University, Adana 01330, Turkey; deryaozduran@yahoo.com (F.D.B.); dozonur@yahoo.com (D.K.); esraulas8280@gmail.com (E.K.); drburcukoseci@gmail.com (B.K.); iremkekec@hotmail.com (İ.K.); mungan@cu.edu.tr (N.O.M.); 2 Department of Dietetics, Cukurova University, Adana 01330, Turkey; ebrucicek94@gmail.com (E.Ç.T.); tugceozakcaoglu@gmail.com (T.K.); semauveyikkk@hotmail.com (S.U.); 3 Department of Social Pediatrics, Cukurova University, Adana 01330, Turkey; adnan_barutcu@hotmail.com; 4 Department of Biostatistics, Cukurova University, Adana 01330, Turkey; nazlitotik.biostat@gmail.com

**Keywords:** phenylketonuria, feeding models, sleep quality

## Abstract

Background: Phenylketonuria (PKU) is an inherited metabolic disorder that requires early diagnosis and strict phenylalanine (Phe)-restricted diet to prevent neurocognitive impairment. Various infant feeding models have been used to achieve optimal metabolic control during early life. The aim of this study was to compare two different feeding models for infants with classical PKU in terms of metabolic control, growth parameters, micronutrient status, the process of introducing complementary foods, and with a particular focus on sleep quality. Methods: In this prospective observational study, 26 infants with classical PKU were followed for 12 months. Patients were assigned to one of two feeding groups: Group-1 received breast milk and Phe-free formula in alternating feeds, while Group-2 received Phe-free formula followed by breastfeeding until satiety. Blood Phe, micronutrient levels and anthropometric measurements were recorded. Sleep quality was evaluated using the Brief Infant Sleep Questionnaire-Revised (BISQ-R). A structured set of parental questions was used to evaluate their experiences during the complementary feeding period. Results: No statistically significant differences were observed between the groups in terms of blood Phe levels, anthropometric measurements, serum levels of iron, ferritin, vitamin-B12, vitamin-D, and zinc. Complementary feeding tolerance were similar across the groups. The BISQ-R analysis revealed no significant differences between the groups. Conclusions: Both feeding models were equally effective in maintaining metabolic control, supporting normal growth, complementary feeding processes, and preserving sleep quality during infancy. These findings suggest that either approach can be adopted based on the preference of the caregiver and the practicality of the clinical setting.

## 1. Introduction

Phenylketonuria (PKU) is the most common inherited metabolic disorder worldwide. It is caused by mutations in the *PAH* gene that lead to a deficiency or complete absence of the phenylalanine hydroxylase enzyme. PKU is inherited in an autosomal-recessive manner. Without treatment, elevated levels of phenylalanine (Phe) cross the blood–brain barrier and disrupt the synthesis of proteins, myelin, and key neurotransmitters. This leads to neurotoxic effects. The cornerstone of this treatment is a phenylalanine-restricted diet. The main aim of this therapy is to maintain blood phenylalanine levels between 120–360 µmol/L, as recommended by European guidelines for managing PKU [1]. The most recently published recommendation in our country proposes setting a three-stage upper limit according to age for PKU treatment. Treatment targets phenylalanine levels of <240 μmol/L for newborns and adolescents, <360 μmol/L for adolescents and adults, and <600 μmol/L for adults [2].

Although dietary treatment remains the gold standard for the management of PKU, various feeding models have been employed despite similarities in dietary content. These include administering a measured amount of expressed breast milk, followed by a phenylalanine-free (Phe-free) formula, offering limited breastfeeding after a Phe-free formula, allowing breastfeeding until the baby is full after a fixed volume of Phe-free formula, and alternating between breast milk and Phe-free formula throughout the day. These practices may vary depending on the country or management center’s approach. A commonly used method involves administering a pre-calculated amount of Phe-free formula, followed by breastfeeding [3,4,5,6,7,8,9]. Using this method, it is believed that the infant will become sufficiently satiated by the Phe-free formula. This results in a reduced appetite during the feeding session, decreased breast milk intake, and, consequently, lower phenylalanine consumption. An alternative feeding approach involves the alternate administration of breast milk and a Phe-free formula during different feedings. With this method, the frequency of breastfeeding was adjusted according to blood phenylalanine levels. There is a lack of data in the literature regarding the long-term effects of these methods on metabolic control.

This study aimed to compare the effects of two different feeding models on the metabolic control, growth parameters, and blood levels of vitamins, minerals, and iron in infants diagnosed with classical PKU. Furthermore, this study is the first to evaluate the potential impact of two different feeding models on factors that directly influence the quality of life, such as sleep patterns and the transition to complementary feeding. To our knowledge, this is the first study in the literature to be conducted exclusively in patients with classical PKU, and it has the largest sample size to date. This study aimed to make meaningful contributions to the clinical management of the most prevalent inherited metabolic disorders worldwide.

## 2. Material and Methods

This prospective observational study was conducted at the Division of Pediatric Metabolism at Çukurova University between May 2021 and April 2023. During the study period, a total of 33 patients were diagnosed with classical PKU. Of these, 26 patients consented to participate in the study and were included in all adherence and outcome analyses. Seven patients did not participate due to parental decision. All patients were term infants with a normal birth weight. The birth order of the infants was recorded and the distribution was similar across the study groups. Newborn screening was performed for all infants within the first 48 h after birth, following oral feeding. A second blood sample was collected on days 5 and 7. The results and the families’ referral to our center typically took place within 10–17 days. The average day of referral was 15.3. The diagnosis was established based on elevated plasma phenylalanine concentrations (>1200 μmol/L), which were detected via newborn screening and confirmed by plasma amino acid analysis performed upon admission. Genetic testing was also performed on all patients and pathogenic mutations in the *PAH* gene consistent with classical PKU were identified, thereby confirming the diagnosis. Ethical approval was obtained from the Ethics Committee of Çukurova University, in accordance with the principles of the Declaration of Helsinki (4 March 2022, Meeting No. 120). Written informed consent was obtained from all parents before their children’s participation. The parents of the 26 infants included in the study were informed about the study design and the randomization of feeding methods prior to enrollment. No parents expressed concerns or objections regarding the assigned feeding strategy, and all agreed to participate. Patients were systematically assigned to one of the two feeding model groups based on the order of admission. According to this method of allocation, the first patient was assigned to group-1, the second to group-2, the third to group-1, and the fourth to group-2. This sequence was maintained throughout the study. In Group-1, breast milk and Phe-free formula were administered alternately during different feeding periods. In Group-2, patients were breastfed until satiety following the administration of a pre-measured Phe-free formula within the same feeding session.

All assessments were carried out during the first year of life, a critical period for infants and parents alike, especially with regard to diagnosis and transition to complementary feeding. Patients were monitored for 12 months. Blood phenylalanine levels were measured weekly using high-performance liquid chromatography (HPLC). At the third and sixth months, laboratory analyses included complete blood count and serum iron, ferritin, vitamin B12, vitamin D, and zinc levels. Anthropometric measurements were recorded at the 3rd, 6th, 9th, and 12th months of age.

All dietary plans were prepared by an experienced dietitian specializing in metabolic disorders. The prescribed dietary intake schedule was determined according to our routine clinical protocol. Parents were thoroughly instructed on the prescribed schedule, including the timing and composition of Phe-free formula and complementary foods, and the importance of strict adherence for optimal metabolic control. Adherence to the prescribed schedule was monitored at each clinical visit through detailed diet records routinely completed by families, which also allowed verification of the actual dietary intakes, including the amounts of phenylalanine, protein, and energy consumed, as well as the number and timing of Phe-free formula and complementary food intakes to assess adherence, feasibility, and provide accurate reporting of daily dietary intakes. After following a washout diet based on their blood phenylalanine levels, all patients were started on a phenylalanine-restricted diet providing 30–40 mg/kg/day phenylalanine, 2.5 g/kg/day protein, and 120 kcal/kg/day energy. All infants in the study were directly breastfed. In Group 1, infants were also fed every 2 h, alternating between one Phe-free formula feed and one direct breastfeeding session. In Group 2, infants were fed every 2 h, with Phe-free formula given first, followed immediately by direct breastfeeding. Between 3 and 6 months of age, the feeding interval was extended to every 3 h for both groups. After 6 months, feeding was provided 8 times over a 24 h period, with four of these feeds consisting of Phe-free formula. The volume of breast milk intake was estimated based on age-appropriate gastric capacity for each infant. Phenylalanine intake from breast milk was calculated accordingly and combined with the precisely measured intake of the Phe-free formula to determine the total daily Phe intake (mg/kg/day). For breast milk, a reference phenylalanine content of 46 mg/100 mL was used in accordance with European guidelines [1]. For Phe-free formulas, the nutritional information on the product labels was used as the reference.

One month after the introduction of complementary feeding, all parents were asked to answer a set of pre-prepared evaluation questions regarding the process. As there is no standardized, validated scale available in the literature to assess complementary feeding intake, these questions were specifically developed for this study, based on the expert opinions of experienced pediatric metabolism specialists working at the same center. The evaluation covered the timing of the introduction of complementary foods, types of food introduced, and amount consumed in each portion. It also covered the infant’s initial reactions to new foods and any potential adverse effects following the introduction of the foods, such as rashes, constipation, diarrhea, vomiting, and irritability. The emotional experiences of parents, including their concerns about nutrition and dietary management, were explored. Changes in feeding patterns, sleep variations, the timing of introducing new foods were also assessed.

The Brief Infant Sleep Questionnaire-Revised (BISQ-R), a validated and reliable tool for assessing infant sleep quality, was administered to the parents of infants in the two feeding model groups to assess sleep quality. A control group of 26 healthy term infants with no known chronic or metabolic diseases was included for the sole purpose of sleep assessment. This group was matched to the study group in terms of age and sex distribution. The sleep questionnaire was administered at the end of the third, sixth and twelfth months. The assessment was initiated at three months of age because newborns typically experience transitional sleep patterns, including irregular cycles and an absence of circadian rhythm differentiation (i.e., a day–night distinction). Conducting the evaluation after the neonatal period enabled a more reliable assessment of sleep patterns to be made once physiological maturation had begun. The sleep questionnaire was an additional procedure performed as part of the study. The variables of the questionnaire included nocturnal sleep onset time, wake-up time, nocturnal sleep duration, daytime sleep duration, number of night awakenings, duration of wakefulness during the night, settling time (latency to falling asleep for the night), method of falling asleep, location of sleep, preferred body position, and parental perception of the child’s sleep.

### Statistical Analyses

The summary statistics of the variables included the mean, standard deviation, and median with 25th and 75th quartiles. Categorical variables were represented as numbers and percentages. The normality of the distribution was established using the Shapiro–Wilk test. The statistical analysis was conducted using a chi-square test, which was employed to compare the categorical variables between the groups. The normality of distribution for continuous variables was confirmed with the Shapiro–Wilk test. For non-normal distributed data, the Kruskal–Wallis test was employed to compare more than two groups. The Mann–Whitney U test was employed for the Bonferroni correction of independent variables. In order to evaluate the change in the measurements obtained in the specified time interval, the Repeated Measurements Analysis or Friedman Test was applied, depending on whether the statistical hypotheses were fulfilled or not. Wilcoxon’s test was employed, followed by the Bonferroni correction for multiple comparisons of paired samples. All statistical analyses were conducted using IBM SPSS 20 (Armonk, NY, USA: IBM Corp.). *p* values less than 0.05 were considered to be statistically significant.

## 3. Results

A total of 26 infants with classical PKU, referred through the national newborn screening program, participated in and completed the study. After achieving blood phenylalanine levels below 360 µmol/L using the initial wash-out diets, the patients were divided into two groups for treatment. Each group comprised 13 participants. Group 1 (*n* = 13) consisted of infants who were breastfed and administered Phe-free formula alternately in separate feedings throughout the day. Group 2 (*n* = 13) included infants who were breastfed until satiety, following the administration of a pre-measured Phe-free formula within the same feeding session. Group 1 had a female-to-male ratio of 8:5, whereas Group 2 had a ratio of 6:7. The mean age at the initiation of dietary treatment was 15.3 days.

At the initiation of dietary treatment, patients in Group 1 received an average of 42.79 ± 10.28 mg/kg/day of phenylalanine, 2.41 ± 0.31 g/kg/day of protein and 120.54 ± 9.90 kcal/kg/day of energy. Group 2 received an average of 43.15 ± 7.45 mg/kg/day of phenylalanine, 2.52 ± 0.17 g/kg/day of protein and 119.85 ± 4.12 kcal/kg/day of energy from their diet. At each clinical visit, analysis of diet records maintained by the parents confirmed that the infants adhered to the prescribed dietary intake schedule. Despite the potential challenges of the feeding regimen, families consistently followed the recommended schedule, providing feeds every 2 h during the first 3 months and every 3 h between 3 and 6 months, and, after the introduction of complementary foods, consuming both Phe-free formula feeds and complementary foods as instructed. After 6 months, infants received a total of eight dietary intakes over 24 h, comprising four Phe-free formula feeds and four complementary food portions. There were no statistically significant differences between the groups in terms of the dietary content (*p* > 0.05).

During the 12-month follow-up period, metabolic control was evaluated. No statistically significant difference was found between the groups in terms of the blood phenylalanine levels. Table 1 shows the mean blood phenylalanine levels in both groups.

**Figure 1:**
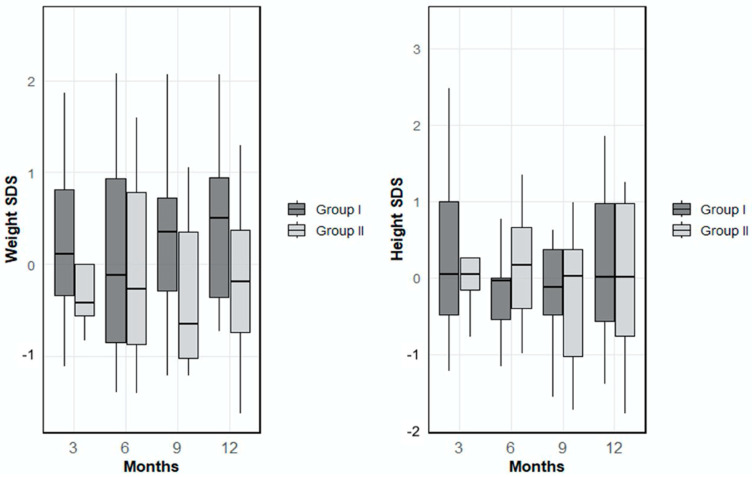
Mean weight and height standard deviation scores (SDS) of Group 1 and Group 2 at 3, 6, 9, and 12 months of age.

Examination of the anthropometric parameters revealed that the mean weight Standard Deviation Score (SDS) in Group 1 was 0.27 ± 0.88 at month 3, 0.17 ± 1.15 at month 6, 0.23 ± 0.95 at month 9 and 0.44 ± 0.90 at month 12. In Group 2, the mean weight SDS values were −0.19 ± 0.75 at month 3, −0.06 ± 1.00 at month 6, −0.17 ± 1.10 at month 9, and −0.13 ± 1.02 at month 12. There were no statistically significant differences between the groups in terms of mean weight SDS values at any time point (*p* > 0.05). When height SDS (Standard Deviation Score) was evaluated, the mean height SDS in Group 1 was 0.43 ± 1.29 at month 3, −0.18 ± 0.78 at month 6, 0.06 ± 1.09 at month 9, and 0.16 ± 0.97 at month 12. In Group 2, the corresponding values at 3, 6, 9, and 12 were 0.19 ± 0.69, 0.17 ± 0.76, −0.26 ± 0.91, and −0.06 ± 0.94, respectively (Figure 1). No statistically significant differences were observed between the groups in height SDS at any of the follow-up time points (*p* > 0.05).

Both groups received standard iron and vitamin supplementation, and differences in serum parameters were evaluated to determine whether they were related to the feeding model. Biochemical parameters, including hemoglobin, hematocrit, serum iron, ferritin, vitamin B12, 25(OH)D, and zinc levels for Group 1 and Group 2, are presented in Table 2. Serum iron and ferritin levels in all patients were within the normal ranges. Two patients in each group had serum vitamin D levels < 20 ng/mL. Two patients from Group 1 and three from Group 2 had serum vitamin B12 levels between 200 and 300 pg/mL. No statistically significant differences were found between the two groups in these parameters (*p* > 0.05).

### 3.1. Complementary Feeding Transition Assessment Results

Complementary feeding practices in both groups were assessed using nine structured parental questions. New foods were introduced every three days, leading to an average of 7–9 different foods by the end of the first month. Feeding schedules were similar in both groups. In Group 1, 53.8% of infants began with fruits, mostly bananas. Intake amounts, parental perceptions of feeding success, and gastrointestinal or behavioral effects varied and are detailed in Table 3. Notably, 30.8% reported increased satiety and well-being, while 23.1% expressed concern about dietary errors. Formula intake decreased in 30.8%, and breastfeeding interest declined in 7.7%. Sleep remained unchanged in most, though 7.7% reported increased night awakenings. In Group 2, 69.2% started with vegetables, primarily potatoes. Feeding responses and related observations, including satiety (46.2%) and reduced breastfeeding interest (23.1%), are presented in Table 3. No decrease in formula intake was noted. Night awakenings increased in 15.4%, while most reported stable sleep. No significant differences were found between the groups in any complementary feeding outcomes (*p* > 0.05) (Table 3).

### 3.2. BISQ-R Questionnaire Results

**Figure 2:**
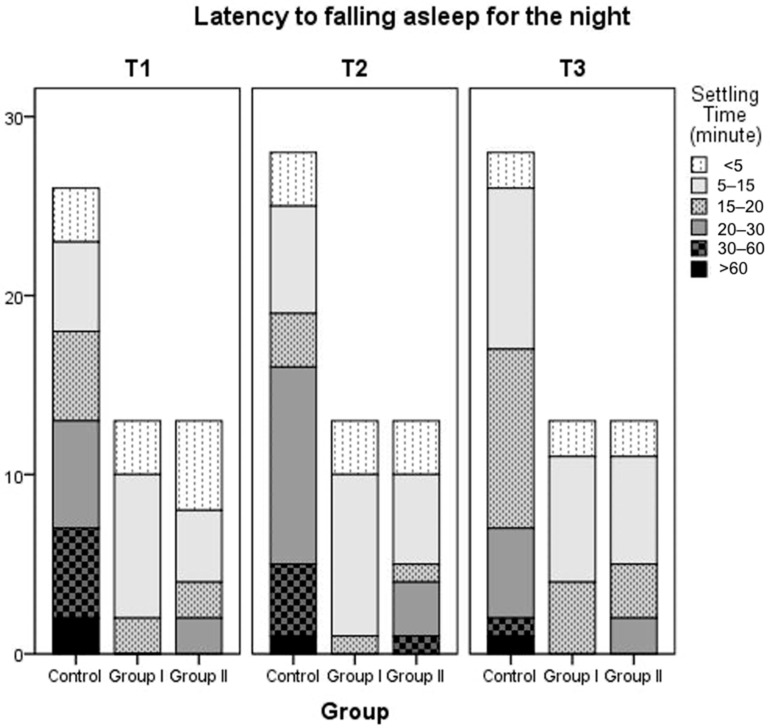
Settling time (latency to falling asleep at night) of patients in Group 1, Group 2, and the control group.

**Figure 3:**
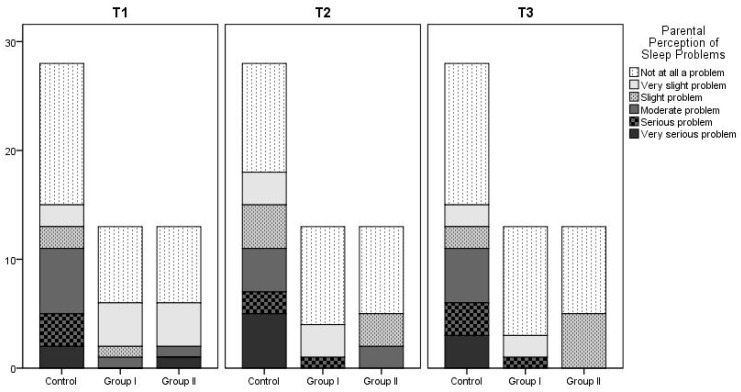
Parental perception of sleep problems in Group 1, Group 2, and the control group at T1 (3rd month), T2 (6th month), and T3 (12th month).

The following questionnaire variables were evaluated: nocturnal sleep onset time, wake-up time, nocturnal sleep duration, daytime sleep duration, number of awakenings at night, duration of wakefulness during the night, latency to falling asleep at night, method of falling asleep, location of sleep, preferred body position, and parental perception of their child’s sleep. No statistically significant differences were found between the groups for any parameter. The mean values and *p*-values for nocturnal sleep duration at 3 (Time 1), 6 (Time 2), and 12 months (Time 3), the number of night-time awakenings, daytime sleep duration, and latency to falling asleep over time and between groups are summarized in Table 4 and Table 5. Latency to falling asleep at night of patients in Group 1, Group 2, and the control group is also summarized in Figure 2. According to the findings regarding parental perceptions of their child’s sleep as a problem, the proportion of parents who reported that they did not consider their child’s sleep a problem increased over time across all groups. There was no statistically significant difference between the three groups. At T1, 7.7% of parents in Group 2 reported sleep problems, but this figure decreased to 0% at T2 and T3. No sleep problems were reported by parents in Group 1 at the 6th and 12th months. Figure 3 shows details.

## 4. Discussion

Phenylketonuria is an inherited metabolic disorder for which early diagnosis and treatment can prevent neurological complications. Diagnosis within the first 15 days of life and prompt treatment are critical for determining the disease course [1]. A multicenter survey was conducted in Europe to evaluate the diagnostic and treatment practices for infants diagnosed with PKU. Data from 95 centers across 21 countries were used for evaluation [10]. A low-phenylalanine diet was initiated within the first 10 days of life in > 60% of the centers. Treatment was started between days 11 and 14 in 31% of the centers, between days 15 and 20 in 6%, and after day 21 in 1% of the centers. In our study, dietary treatment was initiated within the first 20 days of life, further emphasizing the critical importance of early diagnosis and prompt intervention, which may help prevent potential neurodevelopmental sequelae by maintaining the phenylalanine levels within the target range.

Various feeding strategies have been developed for infants diagnosed with PKU to preserve the benefits of breastfeeding while simultaneously achieving optimal metabolic control. A commonly used approach in Europe involves administering a measured amount of Phe-free formula prior to each breastfeeding session, followed by breastfeeding until satiety [10]. As an alternative, some centers alternately administer breast milk and a Phe-free formula throughout the day [11]. Another method commonly used in Europe involves providing a standard infant formula along with a Phe-free formula during a single feeding session [10]. Although this approach is practical, reducing the proportion of the standard formula could affect its taste, potentially causing the infant to refuse to feed and preventing them from receiving breast milk. There is limited data on the effects of implementing these feeding models during the first year of life and their comparison with each other [3,4]. A study by Zuvadelli et al. involving 19 patients with classical PKU showed that metabolic control did not differ despite the use of different feeding methods [12]. This study found that breastfeeding duration was significantly longer for infants who were directly breastfed and received a Phe-free formula before feeding. Metabolic control was maintained adequately in all groups. These findings emphasize the importance of integrating approaches that support continued breastfeeding in clinical nutritional management, wherever possible. Although direct measurement of breastfeeding duration was not performed in our study, we found that metabolic control was similarly maintained in infants receiving breast milk and Phe-free formula, regardless of the same or different feeding session. The monthly average phenylalanine levels indicated that metabolic control was good in both groups, with most patients maintaining phenylalanine levels within the target range of less than 360 µmol/L. (Table 1)

In a large-scale study, patients with PKU on a Phe-free formula showed elevated serum levels of folic acid, vitamin B12, copper, and vitamin E, with adequate vitamin A and zinc levels [13]. Our findings are consistent with these observations. Regardless of feeding type, all patients had normal iron, ferritin, and zinc levels. Of these patients, 84% had normal vitamin D levels and 80.7% had normal vitamin B12 levels. No statistically significant differences were observed between the two groups. These results imply that both feeding models had a similar impact on micronutrient status and that combining breast milk with a Phe-free formula alongside routine vitamin and mineral supplementation was sufficient to fulfill the infants’ micronutrient requirements. Moreover, it can be concluded that optimal metabolic control was achieved during the first year of life, with no negative impact on growth or nutrient intake.

The growth of patients with PKU has been the subject of numerous studies. One Spanish study reported growth retardation in children and adolescents with PKU [14]. Conversely, certain studies have reported that the prevalence of age-adjusted short stature among patients with PKU is relatively low, at approximately 5% [15,16]. Another study evaluated the growth parameters of 33 children with early PKU treatment. These parameters were assessed at one month and two years of age. Growth parameters were normal during the early months, although a significant decline was observed at one year. However, these values reportedly return to normal by the age of two years [17]. In our study, no significant differences were observed in the height and weight SDS values measured between the two groups subjected to different feeding models. Throughout the follow-up period, most patients in both groups maintained weight and height SDS values within the range of −1 to +1. These findings suggest that both feeding models provided adequate nutrition during early life and had a positive effect on growth, as the patients were within the normal growth range for their age and sex.

Although the introduction of complementary feeding followed a broadly similar timeline in both groups, differences were observed in the types of first foods preferred and the infants responses to the process. A survey of parents of children diagnosed with classical PKU in Ireland in 2023 found that more than half (52%) reported significant concerns during the complementary feeding period, primarily related to the complexity of protein calculation and the difficulty in maintaining stable phenylalanine levels [18]. In our study, 23.1% of the parents in Group 1 and 7.7% of those in Group 2 experienced anxiety related to the risk of making dietary errors. When all findings were evaluated together, it was found that patients in Group-2 had greater success in tolerating complementary foods and exhibited a favorable profile in terms of maintaining regular formula intake. In contrast, the positive aspects observed in Group-1 included higher consumption of complementary foods, less reduction in interest in breastfeeding, and a smaller effect of the transition to complementary feeding on sleep. Although such differences were noted between the groups, they were not statistically significant. To enhance the generalizability of these findings and enable a more robust interpretation of the observed differences, future studies should use larger sample sizes and standardized, highly valid assessment tools.

Sleep problems in infants are important for both neurodevelopmental processes and the psychosocial well-being of parents. These issues tend to become more pronounced during periods such as vaccination, the introduction of complementary foods, and illness. Owing to the chronic nature of PKU and its potential to interfere with neurotransmitter synthesis, this disorder may negatively impact sleep regulation [19]. Although the impact of phenylketonuria on sleep regulation has been investigated in previous studies [19,20,21], no study has yet been conducted that specifically examined the quantity and quality of sleep in infants with phenylketonuria. The Brief Infant Sleep Questionnaire-Revised (BISQ-R) is a parent-reported assessment tool used in this study that provides multidimensional data on different aspects of infant sleep. Developed by Sadeh et al., the BISQ-R has been validated using subjective (parental reports) and objective (sleep diaries) data and is now widely used to screen for sleep problems in early childhood [22]. A validation study conducted by Boran et al. (2015) demonstrated that the Turkish version of the BISQ-R is a comprehensible, acceptable, and reliable tool for assessing sleep problems in early childhood [23]. In the same study, infants were classified as “poor sleepers” based on Sadeh’s criteria if they experienced more than three night awakenings, remained awake for more than one hour during the night, or had a total sleep duration of less than nine hours within 24 h. Furthermore, parental perceptions of sleep problems were significantly associated with objective indicators of sleep disturbance. Therefore, the BISQ-R’s ability to assess both objective sleep parameters and parental perception makes it particularly useful during infancy. In our study, the BISQ-R questionnaire was administered to patients in Groups 1 and 2 diagnosed with classical phenylketonuria, as well as to a healthy control group. The sleep patterns of the healthy control group were generally similar to those reported in previous studies [24]. Boran et al. described comparable total and daytime sleep durations in infants in the same age range [24]. This suggests that the sleep data from our control group align reasonably with previous reports and provide a useful reference for interpreting the sleep outcomes of infants with PKU. According to the criteria defined by Sadeh, none of the groups had an average number of night awakenings exceeding three awakenings. In none of the groups was the average duration of nocturnal wakefulness longer than one hour. In Group-2, based on the BISQ-R results from the third month, the mean total sleep duration was found to be 8.5 h; in all other time points and groups, the total sleep duration was nine hours or more. According to the responses to the questionnaire from parents across the three groups, minor differences were observed in the sleep parameters; however, statistical analyses revealed no significant differences (*p* > 0.05).

A study by Gassió et al. involving 32 patients with PKU (16 females and 16 males with a mean age of 12 years) and a control group of 32 healthy individuals found that the prevalence of sleep disturbances was similar in both groups (15.6% in the control group and 12.5% in the PKU group) [19]. No correlation was found between peripheral biomarkers of neurotransmitter synthesis, various phenylalanine-related parameters, and sleep disturbances in patients with PKU. Interestingly, despite melatonin and serotonin deficiencies, the prevalence of sleep disorders in patients with PKU treated early has been reported to be similar to that in the general population [19]. The absence of significant differences between the two groups with different feeding models in our study suggests that the nutritional approach used may have only a limited impact on the quality of sleep. Furthermore, the lack of a notable difference in sleep quality between patients with PKU and those in the control group, coupled with the favorable metabolic control observed in our patients, suggests that the degree of metabolic control may influence the occurrence of sleep disturbances.

## 5. Conclusions

In the classical PKU diet, the most common feeding model for infants involves administering Phe-free formula before breastfeeding. In our study, no statistically significant differences were found between Group 2, who followed this feeding model, and Group 1, who received the Phe-free formula alternately with breastfeeding, in terms of metabolic control, growth, the introduction of complementary foods, serum vitamin and trace element levels, and sleep quality. These findings suggest that the two most common feeding approaches can be chosen according to patient compliance and maternal preferences. Furthermore, our study found that both objective sleep parameters and parental perception were consistent with existing normative data for healthy Turkish children, suggesting that well-controlled PKU may not significantly affect sleep during the first year of life.

A notable finding of our study was that parents in both groups fully adhered to the diet. This finding further supports the idea that weekly consultations with physicians and dietitians enhance dietary compliance, when feasible.

## Figures and Tables

**Table 1:** Monthly Mean Plasma Phenylalanine Levels (µmol/L) Between Group 1 and Group 2.

Month	Group 1	Group 2	*p*-Value	*p*-Value
After wash-out	156 (18–336)	36 (12–90)	0.134	Time 0.383 Group * Time 0.624
Month 1	132 (30–204)	96 (12–228)	0.810	
Month 2	78 (30–156)	138 (48–252)	0.319	
Month 3	66 (42–132)	138 (54–168)	0.152	
Month 4	60 (30–180)	144 (18–288)	0.378	
Month 5	84 (24–234)	138 (66–312)	0.362	
Month 6	114 (48–210)	318 (30–522)	0.139	
Month 7	**90 (30–186)**	**108 (30–330)**	**0.611**	
Month 8	**84 (36–186)**	**78 (42–216)**	**0.863**	
Month 9	**96 (24–312)**	**138 (48–630)**	**0.448**	
Month 10	**186 (66–378)**	**150 (42–252)**	**0.437**	
Month 11	**108 (54–246)**	**240 (66–330)**	**0.204**	
Month 12	**138 (42–204)**	**288 (24–396)**	**0.479**	

Values are expressed as median (min–max). Phenylalanine concentrations are presented in µmol/L. Phenylalanine concentrations measured after the initiation of complementary feeding are presented in bold. * Interaction between group and time

**Table 2:** Comparison of Serum Hematological and Micronutrient Parameters Between Groups.

Serum Parameter	Group 1	Group 2	*p*-Value
**Hemoglobin (g/dL)**	11.2 ± 1.3	10.9 ± 0.6	>0.05
**Hematocrit (%)**	32.6 ± 5.0	32.0 ± 1.9	>0.05
**Serum Iron (µg/dL)**	50.2 ± 24.8	48.8 ± 18.0	>0.05
**Serum Ferritin (ng/mL)**	173.5 ± 257.7	236.7 ± 262.2	>0.05
**Vitamin B12 (pg/mL)**	576.8 ± 269.2	452.0 ± 178.2	>0.05
**25(OH)D (ng/mL)**	31.7 ± 9.5	34.5 ± 12.9	>0.05
**Serum Zinc (µg/dL)**	576.8 ± 269.2	452.0 ± 178.2	>0.05

Values are presented as mean ± standard deviation (SD).

**Table 3:** Comparison of Complementary Feeding Responses Between the Groups.

Questions ^a^	Group 1	Group 2	*p* ^b^
**How much of the calculated portion of complementary food did the baby consume?**			0.769
Consumed the entire portion	3 (23.1%)	1 (7.7%)	
Consumed half	4 (30.8%)	3 (23.1%)	
Consumed only a small amount	5 (38.5%)	8 (61.5%)	
Could not eat at all	1 (7.7%)	1 (7.7%)	
**How did the baby respond to complementary food?**			>0.999
Rejected/spat out/vomited	4 (30.8%)	4 (30.8%)	
Tried but couldn’t finish	6 (46.2%)	7 (53.8%)	
Ate very well	1 (7.7%)	1 (7.7%)	
Wanted more	2 (15.4%)	1 (7.7%)	
**Problems observed after introduction of complementary food**			0.088
Constipation	4 (30.8%)	1 (7.7%)	
Diarrhea	1 (7.7%)	0 (0%)	
Vomiting	1 (7.7%)	0 (0%)	
None observed	7 (53.8%)	12 (92.3%)	
**How did you feel during the complementary feeding period?**			0.414
Felt the baby was more satiated	4 (30.8%)	6 (46.2%)	
Worried about making dietary mistakes	3 (23.1%)	1 (7.7%)	
Felt better with addition of complementary foods	4 (30.8%)	6 (46.2%)	
Other	2 (15.4%)	0 (0%)	
**After starting complementary foods**			0.160
Formula intake decreased	4 (30.8%)	0 (0%)	
Consumed formula more easily	2 (15.4%)	3 (23.1%)	
Showed less interest in breast milk; harder to feed	1 (7.7%)	0 (0%)	
Showed more interest in breast milk; easier to feed	1 (7.7%)	1 (7.7%)	
No change observed	5 (38.5%)	9 (69.2%)	
**Did your baby’s sleep pattern change after starting complementary food?**			0.720
Yes, woke up more frequently	1 (7.7%)	2 (15.4%)	
Yes, woke up less frequently	2 (15.4%)	3 (23.1%)	
No change	10 (76.9%)	8 (61.5%)	

^a^ Each question was asked as a separate item in the structured questionnaire. ^b^ *p*-values calculated using Fisher’s exact test. Data are presented as numbers (percentage).

**Table 4:** Comparison of BISQ-R Sleep Questionnaire Results Between Groups and Over Time.

		Group				
Sleep Parameter	Time	Control Group	Group 1	Group 2	*p*	*p*
**Nocturnal sleep duration (hours)**	**T1**	7.3 ± 2.6 7 (6–10)	8.6 ± 2.5 9 (7–10.5)	8.1 ± 2.9 8.5 (7.3–10)	0.289	Time 0.048 Group * Time 0.383
	**T2**	8 ± 2.1 8 (6.3–9.8)	8.7 ± 1.6 9 (7.5–10)	8.9 ± 1 9 (8.5–9)	0.179	
	**T3**	9 ± 1.9 ^φ^ 10 (7–10)	8.6 ± 1.6 9 (7.5–9.5)	9.3 ± 0.6 9 (9–9.5)	0.452	
	*p*	0.026	0.819	0.338		
**Daytime sleep duration (minutes)**	**T1**	175.2 ± 113.3 150 (105–240)	226.2 ± 141.6 180 (105–330)	234.6 ± 125.7 170 (135–360)	0.310	Time 0.001 Group * Time 0.210
	**T2**	153.9 ± 51.1 150 (120–180)	197.7 ± 125.5 130 (120–300)	215.4 ± 71.6 180 (170–285)	0.058	
	**T3**	149.6 ± 67.5 150 (120–180)	156.9 ± 66.6 120 (120–170)	116.9 ± 45.5 ^†^ 120 (80–150)	0.151	
	*p*	0.336	0.424	0.009		
**Number of night awakenings**	**T1**	3 (1.3–3)	3 (2–4)	2 (1.5–3)	0.786	Time 0.096 Group * Time 0.274
	**T2**	3 (2–3.8)	2 (1.5–3.5)	2 (2–3)	0.440	
	**T3**	2.5 (1.3–4)	1 (1–3)	1 (1–2) ^φ, ϒ^	0.041	
	*p*	0.304	0.062	0.008		
**Duration of nocturnal wakefulness (minutes)**	**T1**	25.5 ± 19.5 20 (11.3–30)	14 ± 4.2 15 (10–17.5)	25 ± 36.2 10 (10–22.5)	-	-
	**T2**	20.3 ± 12.5 17.5 (10–30)	10 ± 0 10 (10–10)	10 ± 0 10 (10–10)	-	-
	**T3**	17.4 ± 15.5 10 (10–15)	10 ± 0 10 (10–10)	10 ± 0 10 (10–10)	-	-
	*p*	-	-	-		

T1: Time 1 (3rd month), T2: Time 2 (6th month), T3: Time 3 (12th month); ^φ^ *p* < 0.05 between T1; ^†^ *p* < 0.05 between T2; ^ϒ^ *p* < 0.05 between control group. Data were expressed as mean ± standard deviation with median (IQR_25– 75_) or just median (IQR_25– 75_). * Interaction between group and time.

**Table 5:** Sleep Latency of Patients.

	Sleep Latency	Control Group	Group 1	Group 2
**T1**	<5 min	3 (11.5)	3 (23.1)	5 (38.5)
	5–15 min	5 (19.2)	8 (61.5)	4 (30.8)
	15–20 min	5 (19.2)	2 (15.4)	2 (15.4)
	20–30 min	6 (23.1)	0 (0.0)	2 (15.4)
	30–60 min	5 (19.2)	0 (0.0)	0 (0.0)
	>60 min	2 (7.7)	0 (0.0)	0 (0.0)
**T2**	<5 min	3 (10.7)	3 (23.1)	3 (23.1)
	5–15 min	6 (21.4)	9 (69.2)	5 (38.5)
	15–20 min	3 (10.7)	1 (7.7)	1 (7.7)
	20–30 min	11 (39.3)	0 (0.0)	3 (23.1)
	30–60 min	4 (14.3)	0 (0.0)	1 (7.7)
	>60 min	1 (3.6)	0 (0.0)	0 (0.0)
**T3**	<5 min	2 (7.1)	2 (15.4)	2 (15.4)
	5–15 min	9 (32.1)	7 (53.8)	6 (46.2)
	15–20 min	10 (35.7)	4 (30.8)	3 (23.1)
	20–30 min	5 (17.9)	0 (0.0)	2 (15.4)
	30–60 min	1 (3.6)	0 (0.0)	0 (0.0)
	>60 min	1 (3.6)	0 (0.0)	0 (0.0)

Data were expressed as numbers (%).

## Data Availability

The raw data supporting the conclusions of this article will be made available by the authors on request.
